# Regulated CRISPR Modules Exploit a Dual Defense Strategy of Restriction and Abortive Infection in a Model of Prokaryote-Phage Coevolution

**DOI:** 10.1371/journal.pcbi.1004603

**Published:** 2015-11-06

**Authors:** M. Senthil Kumar, Joshua B. Plotkin, Sridhar Hannenhalli

**Affiliations:** 1 Graduate Program in Bioinformatics, University of Maryland, College Park, Maryland, United States of America; 2 Center for Bioinformatics and Computational Biology, University of Maryland, College Park, Maryland, United States of America; 3 Department of Biology, University of Pennsylvania, Philadelphia, Pennsylvania, United States of America; National Center for Biotechnology Information, UNITED STATES

## Abstract

CRISPRs offer adaptive immunity in prokaryotes by acquiring genomic fragments from infecting phage and subsequently exploiting them for phage restriction via an RNAi-like mechanism. Here, we develop and analyze a dynamical model of CRISPR-mediated prokaryote-phage coevolution that incorporates classical CRISPR kinetics along with the recently discovered infection-induced activation and autoimmunity side effects. Our analyses reveal two striking characteristics of the CRISPR defense strategy: that both restriction and abortive infections operate during coevolution with phages, driving phages to much lower densities than possible with restriction alone, and that CRISPR maintenance is determined by a key dimensionless combination of parameters, which upper bounds the activation level of CRISPRs in uninfected populations. We contrast these qualitative observations with experimental data on CRISPR kinetics, which offer insight into the spacer deletion mechanism and the observed low CRISPR prevalence in clinical isolates. More generally, we exploit numerical simulations to delineate four regimes of CRISPR dynamics in terms of its host, kinetic, and regulatory parameters.

## Author Summary

To counteract viral infections, bacteria and archaea have evolved a variety of defense systems. These can broadly be classified into either restriction or suicide mechanisms. The former enforces nicks in the invading DNA making it unusable for production of further infectious particles; the latter, by contrast, induces cell death whereby an infected cell activates specific host suicidal pathways that are otherwise strongly repressed, thus inhibiting further infection. Examples of the former class include *restriction-modification* (R-M) and the recently discovered CRISPR systems, while the latter class includes a variety of *toxin/anti-toxin* systems. CRISPRs, in contrast to R-Ms, adapt to target viral genomes by updating the database of target sites they recognize. The adverse side effect of such a mechanism, however, is that CRISPRs can target the host genome itself resulting in undesirable cell death (autoimmunity). The recent discovery of infection-induced activation of CRISPR systems suggests that these negative side effects may be limited to periods of infection. This led us to hypothesize that such regulatory control—similar to abortive infection mechanisms—can be advantageous by limiting viral spread through suicide of infected cells. To test this hypothesis, we mathematically model CRISPR induced prokaryote-phage coevolutionary dynamics in the presence of infection-regulated CRISPR activity. Our results indicate that, except in limited growth rates, regulated CRISPRs exploit both autoimmunity and target restriction and can therefore be considered a hybrid class that leverages both restriction and suicide mechanisms to limit phage infection.

## Introduction

Prokaryotes have evolved diverse molecular defense systems over billions of years of co-evolution with phages [1,2]. Clustered Regularly Interspersed Palindromic Repeats (CRISPRs), found in roughly 40% of sequenced bacteria and 90% of archaea, are peculiar in that they confer adaptive immunity against invading phages [3–6]. CRISPR, as a defense mechanism, works via targeted acquisition of 26–72bp fragments (called protospacers) from the target DNA, and subsequently use of acquired fragments (spacers) for target restriction through an RNAi-like mechanism [7,8]. Acquisition events appear to concentrate around short 2–5bp motifs (protospacer adjacent motifs, or PAMs) in the target DNA [4,9,10]. CRISPR loci are organized as cassettes in which short repeats interleave spacers, and are located adjacent to highly diverse genes that code for the CRISPR associated protein machinery [4,11].

Intriguingly, in addition to acquiring phage fragments, CRISPR systems can also acquire spacers from the host genome. This has been experimentally demonstrated in two model systems: first, selective induction of the acquisition machinery (in the absence of interference) in laboratory strains of *Escherichia coli* resulted in the accumulation of a large number of self-targeting spacers [12]; second, abolition of interference activity (and not the acquisition machinery) in wild type *Streptococcus thermophilus* resulted in unbiased acquisitions of self-targeting spacers alongside phage-targeting spacers [13]. However, a large-scale survey of CRISPR cassettes in microbial genomes identified that only about 0.4% of the spacers are self-targeting, which, considering the relative size of prokaryotic genomes over phages, suggests some mechanism of selection against self-targeting spacers, perhaps to avoid autoimmunity [14–16]. Indeed, directed experiments have conclusively shown that self-targeting can result in severe lethality [9,17–21].

We therefore face a conundrum: how do prokaryotes maintain functional CRISPR systems [22]? Despite the conceptual similarities with restriction-modification systems that avoids autoimmunity by methylating the host genomes’ target restriction sites [23], no analogous genome wide self- vs. non-self-discrimination (SND) mechanism is known for CRISPR systems. In fact, as noted above, the evidence thus far suggests that an efficient SND may not exist (The SND mechanism described by Marrafini and Sontheimer explains the evasion of self-destruction of CRISPR locus only and does not confer genome wide protection [24]). But there are other routes to avoiding autoimmunity. Toxin/anti-toxin or abortive infection systems restrict the scope of autoimmunity to infected populations via infection-induced activation [25]. Indeed, upregulation of CRISPRs upon phage infection has been demonstrated experimentally [26–28]. This makes it possible that the accumulated self-targeting spacers may function as “toxins”, which can be activated upon infection. We therefore address the following two questions in this study:

1. Does infection-induced activation allow CRISPRs to function as an abortive infection (ABI) system? If so, what is the relative contribution of ABI in determining coevolving host and phage densities?
2. If CRISPR suppression in uninfected host populations is required to avoid host extinction, how strong should this suppression be?

Clearly, the answers to these questions depend on key ecological and CRISPR kinetic parameters. For instance, while CRISPRs are highly active against phages in wild type *S*. *thermophilus* (a lactic acid bacteria widely used in industrial production of cheese) [9], artificial induction is essential to activate the system in *E*.*coli* [29]. To this end, we develop and analyze a dynamical model that integrates prokaryote-phage coevolutionary dynamics, with regulated, infection-induced CRISPR acquisition and interference activity. Several models of CRISPR-mediated prokaryote-phage coevolutionary dynamics have been previously reported [20,30–35]. While refs. [33–35] account for an abstract CRISPR-associated cost, they do not include the specifics of autoimmunity kinetics/the regulatory aspect of CRISPRs. The model we develop here is detailed enough to incorporate the adaptive aspects of CRISPR, and general enough to allow intuitive (analytic) interpretations of the resulting qualitatively distinct steady states. We interrogate the model using simulations and bifurcation analyses, and we find that as a function of key host, ecological, and CRISPR evolutionary parameters, the operational behavior of CRISPRs (and the resulting host densities) decomposes into four qualitatively distinct regimes. In those regimes where CRISPR is advantageous to the host, both restriction and abortive infection operate; the latter dominates restriction in SND absence. Crucially, CRISPR maintenance is determined by an upper bound on the activation level of CRISPRs in uninfected populations. This critical limit of activation—beyond which host extinction is inevitable—is determined by a simple dimensionless combination of parameters. We compare the current experimental data on CRISPR kinetics with these qualitative observations, which helps to explain the spacer deletion mechanism and absence of CRISPR activity in highly virulent and multi-drug resistant clinical isolates.

## Results

### Behavior of a simple prokaryotic immune system with regulated autoimmunity

Before proceeding to model the complexity of CRISPR dynamics in general, we start by considering the case of a simple prokaryotic immune system with regulated autoimmunity. The goal here is to analyze the influences of the regulation, immunity and autoimmunity on the resulting coevolutionary dynamics.

**Fig 1:**
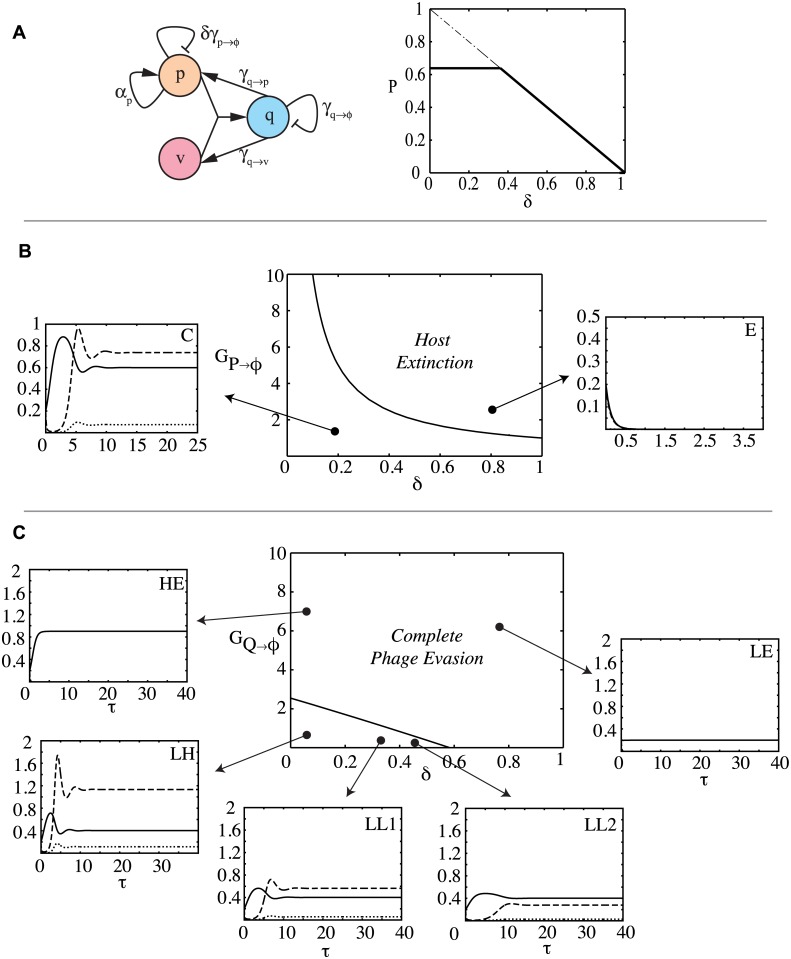
Bifurcation analysis of a simple model of a prokaryotic immune system with regulated autoimmunity side effect. (A) *p*, *q* and *v* denote densities of uninfected, infected cells, and phage respectively. *q* undergoes autoimmunity at a rate of *γ*_*q*→*ϕ*_, while *p* undergoes autoimmunity at a suppressed rate determined by *δγ*_*p*→*ϕ*_. The second figure shows the bifurcation behavior of the free cell densities with respect to the control parameter *δ*, beyond a certain critical value of which one of the steady states vanishes. (B,C) Two-parameter bifurcation diagram revealing coexistence (C) and host extinction (E). Each plot instance is denoted by a tuple <AB> where A and B can indicate low (L) or high (H) values or extinction (E) of prokaryote (free—*solid*, infected—*dotted*) and phage (*dashed*) respectively. *G*_*P*→*ϕ*_ and *G*_*Q*→*ϕ*_ denote the rescaled free cell autoimmunity rate and infected cell autoimmunity (abortive infection) rates respectively. High values of *G*_*Q*→*ϕ*_ lead to complete phage evasion. Parameter values *α*_*p*_ = 1 *hr* ^−1^, Φ = 10^8^ *cells ml* ^−1^, *γ*_*v*_ = 5 *hr* ^−1^, *α*_*v*_ = 50, *α*_*q*_ = 5×10^−9^ *ml phage* ^−1^ *hr* ^−1^.

Fig 1 illustrates a simple coevolutionary model in which the immune system, apart from conferring immunity, also induces autoimmunity that is regulated in a cell state (infected / uninfected) specific manner. Dynamic variables are denoted with Roman letters, and parameters are denoted with Greek symbols. Any parameter associated with production of an item *i* is denoted as *α*_*i*_ and that with its degradation is denoted by *γ*_*i*_. Free cells (*p*), grow exponentially at a rate of *α*_*p*_, under a carrying capacity constraint of Φ_*p*_. Phages (*v*) infect free cells to produce infected cells at a rate of *α*_*q*_. Infected cells can lyse to release phages at a rate of *γ*_*q*→*v*_ or undergo immunity to become a free cell at a rate of *γ*_*q*→*p*_, or undergo autoimmunity at a rate of *γ*_*q*→*ϕ*_. Free cells undergo autoimmunity at a suppressed rate of *δγ*_*p*→*ϕ*_, (0 ≤ *δ* ≤ 1). Note *γ*_*p*→*ϕ*_ need not necessarily equal *γ*_*q*→*ϕ*_, for reasons that will become clear later when we discuss the detailed CRISPR model. The condition *δ* = 0 implies complete repression of autoimmunity in free cells, whereas *δ* = 1 indicates no difference in repression across the two cell states. The burst size of phages is *α*_*v*_. Phages also die at a rate of *γ*_*v*_. Table 1 describes the variables and model parameters.

**Table 1:** Descriptions of variables and parameters in model 1.

Variable	Description	Units
*p*, *q*	Cell Densities	*cells ml* ^−1^
*v*	Phage Density	*phages ml* ^−1^
*α*_*p*_	Reproduction Rate	*hr* ^−1^
*α*_*q*_	Adsorption rate	*ml phage* ^−1^ *hr* ^−1^
*γ*_*p*→*ϕ*_, *γ*_*q*→*ϕ*_, *γ*_*q*→*p*_, *γ*_*q*→*v*_	Autoimmunity, Immunity and lysis rates	*hr* ^−1^
*γ*_*v*_	Phage deactivation rate	*hr* ^−1^
*α*_*v*_	Phage burst size	
*δ*	Regulation parameter	

The dynamical equations for this model can be written as:

$$\begin{array}{l} \dot{p}=\alpha_{p}p\left(1-\frac{p+q}{\Phi_{p}}\right)-\delta \gamma_{p\to \phi }p-\alpha_{q}pv+\gamma_{q\to p}q\\ \dot{q}=\alpha_{q}pv-\left(\gamma_{q\to \phi }+\gamma_{q\to p}+\gamma_{q\to v}\right)q\\ \dot{v}=\alpha_{v}\gamma_{q\to v}q-\gamma_{v}v-\alpha_{q}pv \end{array}$$ (1)

Measuring all the state variables in units of Φ_*p*_, and time in units of *τ* = [*α*_*q*_Φ_*p*_]^−1^ *t*, and denoting all the transformed variables and parameters with their corresponding Roman alphabets, we obtain:

$$\begin{array}{l} \dot{P}=A_{P}P(1-P-Q)-\delta G_{P\to \phi }P+G_{Q\to P}Q-PV\\ \dot{Q}=PV-\left(G_{Q\to \phi }+G_{Q\to P}+G_{Q\to V}\right)Q\\ \dot{V}=\alpha_{v}G_{Q\to V}Q-G_{V}V-PV \end{array}$$ (2)

We can study the influence of regulation (determined by the parameter *δ*), immunity and autoimmunity rates (*G*_*Q*→*V*_, *G*_*Q*→*ϕ*_ and *G*_*P*→*ϕ*_) on the above dynamical system using a bifurcation analysis. These results are summarized in Fig 1. Fig 1A shows that, as a function of *δ*, two fixed points collide at a critical value of *δ* (which we denote by *δ*_1_), beyond which one of them ceases to exist. Fig 1B shows that in the (*δ*,*G*_*P*→Φ_) space, beyond a critical curve that falls roughly as $G_{P\to \Phi }^{-1}$, hosts go extinct. Fig 1C reveals in the (*δ*,*G*_*Q*→Φ_) space, beyond a line of critical points, phages go extinct. Behavior in the (*δ*,*G*_*Q*→*P*_) space is similar. We provide an analytical treatment below.

Bifurcations occur when the number of fixed points or their stability properties change in response to a dynamical parameter. Our system can approach three qualitatively distinct steady states: the first corresponds to host extinction, which we denote by $E^{*}=\left(P_{e}^{*},Q_{e}^{*},V_{e}^{*}\right)=\left(0,0,0\right)$. The second corresponds to a phage free system, which occurs with pure cultures where phages have not been introduced, or when hosts completely evade phage infection, which we denote by $F^{*}=\left(P_{f}^{*},Q_{f}^{*},V_{f}^{*}\right)=\left(P_{f}^{*},0,0\right)$. The third corresponds to the case of prokaryote-phage coexistence, which we denote by $C^{*}=\left(P_{c}^{*},Q_{c}^{*},V_{c}^{*}\right)$.

In the phage free situation, the system evolves along the curve $\dot{P}=A_{P}P(1-P)-\delta G_{P\to \phi }P$, towards the fixed point $P_{f}^{*}=1-\frac{\delta G_{P\to \phi }}{A_{P}}$. Non-extinction/positivity condition on this expression reveals a criticality condition on *δ* for maintenance of hosts carrying our simple immune system in the phage free case: $\delta <\frac{A_{P}}{G_{P\to \phi }}=\delta_{1}$. This is precisely the curve mapped out in Fig 1B beyond which the hosts go extinct; when *δ* = *δ*_1_, F* = E*, and when *δ* > δ_1_, F* is infeasible. Hence, as long as the immune system (with an autoimmunity side effect) is suppressed below a critical nondimensional ratio of the free cell reproduction rate to that of its autoimmunity potential, the phage free steady state is feasible.

The non-trivial fixed point for the case of coexistence, C*, is given by:

$$\begin{array}{l} P_{c}^{*}=\frac{G_{V}}{\frac{\alpha_{v}}{\left(1+\underset{\text{immune advantage}}{\underset{︸}{\frac{G_{Q\to \phi }+G_{Q\to P}}{G_{Q\to V}}}}\right)}-1}\\ V_{c}^{*}=\frac{A_{P}(1-P_{c}^{*})-\delta G_{P\to \phi }}{A_{P}P_{c}^{*}+\frac{G_{Q\to \phi }+G_{Q\to P}}{G_{Q\to V}}}\\ Q_{c}^{*}=\frac{P_{c}^{*}V_{c}^{*}}{G_{Q}} \end{array}$$ (3)

Here *G*_*Q*_ = (*G*_*Q*→*ϕ*_ + *G*_*Q*→*P*_ + *G*_*Q*→*V*_) denotes the overall removal rate of infected cells. In this coexistence regime, the steady state expression for $P_{c}^{*}$ decomposes into the two parts: steady-state value when the dynamics is phage limiting and the advantage offered by the immune system in overcoming phage lysis. This advantage is given by the ratio of the sum of immunity and *autoimmunity* rates conferred by the immune system in *infected* cells to that of the phage specific lysis rates. Thus inducing autoimmunity, alongside immunity, in infected cells (abortive infection) is beneficial to the prokaryotic population when coevolving with phages. As is the case with predator-prey models, $P_{c}^{*}$ is independent of the cell’s own growth rate [36], and is completely determined by the immunity and autoimmunity parameters, along with the phage specific parameters. Furthermore, positivity conditions on the steady state values yields the feasibility conditions for the existence of this steady state: $\left(0<P_{c}*<1\right)$, and (0 ≤ *δ* < *δ*_2_) with $\delta_{2}=\frac{A_{P}(1-P_{c}^{*})}{G_{P\to \Phi }}$ (as $V_{c}^{*}\leq 0$ otherwise), giving us a tighter constraint on *δ* for coexistence. Notice that *δ*_2_ < *δ*_1_. So regardless of the presence or absence of phages, a free cell autoimmunity suppression level of *δ* < *δ*_1_ is required for the population to avoid losing the immune system altogether.

When free cells completely repress the immune system (*δ* = 0), or when there is no autoimmunity (*G*_*Q*→*ϕ*_ = 0), $V_{c}^{*}$ and $Q_{c}^{*}$ achieve their maximum values. As *δ*→*δ*_2_, the values of $V_{c}^{*}$ and $Q_{c}^{*}$ are reduced progressively. The form of these equillibria implies that by increasing the net autoimmunity rate in free cells, lower net viral abundance is achieved. However, by doing so the range of *δ* that supports coexistence is narrowed. When *δ* > *δ*_2_, the coexistence steady state C* is infeasible, and the system operates in the phage free regime, at which point, the condition *δ* < *δ*_1_ has to be satisfied to avoid host extinction. The bifurcation analysis in Fig 1A maps this behavior: C* continues to be stable until *δ* < *δ*_2_, whereas beyond *δ*_2_ the otherwise unstable F* becomes stable (stability of the steady states ascertained by the *Routh-Horwitz* criteria [36]).

To analyze the influence of *abortive infection* on coevolution, we produced a two-parameter bifurcation diagram for the (*δ*,*G*_*Q*→*ϕ*_) space (Fig 1C). Two distinct regimes are clear: a coexistence regime, and a regime where hosts evade phages. A third regime corresponding to host extinction also occurs for autoimmunity suppression exceeding the value *δ*_1_ (for the parameters in this figure, it occurs along the line *δ* = 1). The bifurcation diagrams are similar for a variety of other parameter combinations tested. Coexistence occurs for low values of *G*_*Q*→*ϕ*_, and are progressively lost as *δ* is increased. We can trace the line of critical points analytically as follows. Recall that the switch from coexistence to phage evasion is principally determined by the equality $\delta =\delta_{2}=\frac{A_{P}(1-P_{c}^{*})}{G_{P\to \phi }}$. If we let *G*_*Q*_ = (*G*_*Q*→*V*_ + *G*_*Q*→*P*_ + *G*_*Q*→Φ_) and substituting for $P_{c}^{*}$, we obtain $1-\delta \frac{G_{P\to \Phi }}{A_{P}}=\frac{G_{V}}{\frac{\alpha_{v}G_{Q\to V}}{G_{Q}}-1}$. When $\frac{\alpha_{v}G_{Q\to V}}{G_{Q}}>>1$, as a function of *G*_*Q*→*ϕ*_ and *δ*, this condition spans the line:

$$\frac{\delta }{K_{1}}+\frac{G_{Q\to \phi }}{K_{2}}=1$$ (4)

where the intercepts are given by $K_{1}=\frac{A_{P}}{G_{P\to \phi }}\left[1-\frac{G_{V}}{\alpha_{v}}\left(1+\frac{G_{Q\to P}}{G_{Q\to V}}\right)\right]$ and $K_{2}=G_{Q\to V}\left[\frac{\alpha_{v}}{G_{V}}-\left(1+\frac{G_{Q\to P}}{G_{Q\to V}}\right)\right]$. For the parameters in Fig 1C, Routh-Horwitz criteria [36] reveals that the achieved C* values are stable. Beyond this boundary, coexistence is infeasible, and cells assume a density determined completely by *δ*, and independent of *G*_*Q*→*ϕ*_: $P_{f}^{*}=1-\frac{\delta A_{P}}{G_{P\to \phi }}$. Clearly, both *K*_1_ and *K*_2_ are reduced with increasing values of *G*_*Q*→*P*_ (immune rate), the net effect being reduction of the area under the line resulting in loss of coexistence. To map the influence of *immunity*, one can similarly establish the critical line determining the boundary of coexistence explicitly as a function of (*δ*,*G*_*Q*→*P*_).

In summary, our bifurcation analysis of this simple model (i) reveals the precise regimes for the three possible fates of a prokaryotic immune system with regulated autoimmunity (complete evasion of phages, coexistence with phages, or extinction) (ii) shows that infected cell autoimmunity (alongside restriction) is beneficial to the prokaryotic population, and (iii) reveals a strict limit on the free cell autoimmunity levels above which host extinction occurs.

Perhaps the most characteristic feature of CRISPRs is their adaptive ability for continued novelty resulting from spacer acquisitions and deletions. The model above does not incorporate spacer turnover kinetics or its regulation. Neither does it allow us to explicitly determine the influence of host protospacer levels on the interval of autoimmunity regulation 0 ≤ *δ* < *δ*_1_; the larger this window, the higher the cellular tolerance for CRISPRs.

We will therefore proceed to incorporate CRISPR specific reactions into the simple model described above. We will show that (i) the simple model arises as a particular limit of a more general model, and (ii) by thwarting the accumulation of self-targeting spacers through an SND (whose existence/absence is hard to ascertain from existing data), and/or through a highly active spacer deletion mechanism, the range of free-cell CRISPR activity levels, *δ*, is widened. Furthermore, the general model will reveal other idiosyncratic features of CRISPR and its maintenance in populations over ecological time scales.

### A detailed model for CRISPRs incorporating their adaptive ability and regulation

**Fig 2:**
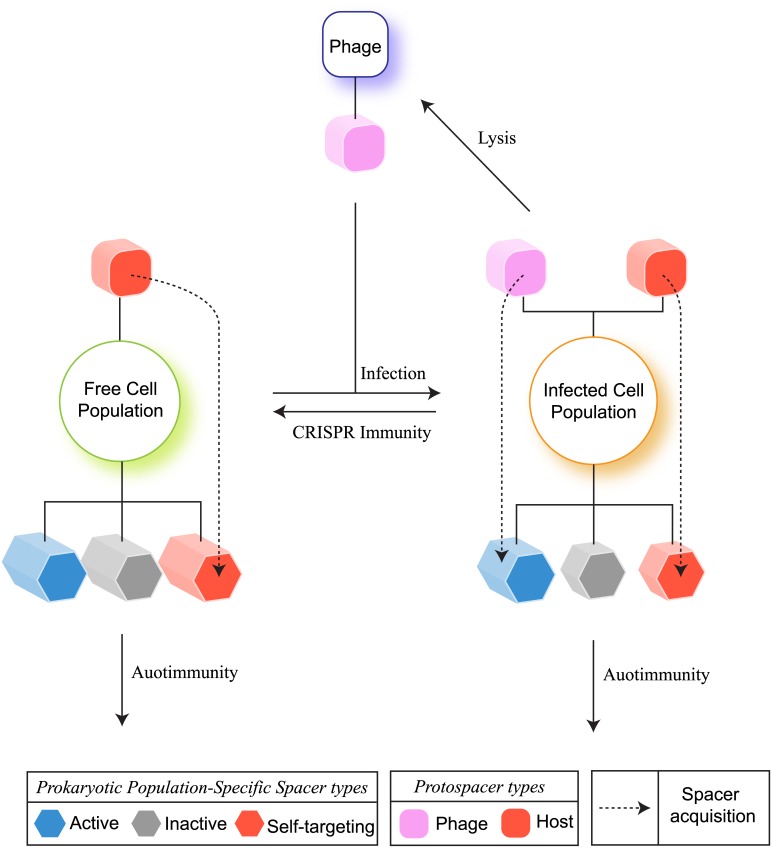
A detailed model of CRISPR dynamics. The infected cell population (and its associated CRISPR spacer content) is created from the growing free cell population (and its corresponding CRISPR content) through phage infections. The overall CRISPR spacer content in each cell population is abstractly partitioned into *active*, *inactive* and *self-targeting*. Active spacers elicit phage restriction, while self-targeting spacers cause cell death (autoimmunity). While both the free and infected cell populations have genomic protospacers that contribute to the creation of self-targeting spacers, only the infected cell population has access to the released phage protospacers for the creation of active spacer content. At any given time, the CRISPR induced rate of immunity for an infected cell is proportional to its per capita quota of active spacer content associated with the population at that time. Similarly, we use the corresponding self-targeting spacer content to define the rates of autoimmunity for both the infected and free cell populations. In our equations, we directly model these per capita quotas. Thus the rates of CRISPR induced immunity and autoimmunity for a cell population are reflective of its associated spacer content at any given time, which in turn is determined by the kinetics of CRISPR and prokaryote-phage interaction.

In this section we develop a more detailed model of CRISPR dynamics, which generalizes the simple model discussed above. Our modeling strategy in this section (see Fig 2) is intermediate to models that fix a constant rate of immunity (as in [30]) and agent-based models that describe strain-specific immunity (as in [32]). Briefly, we track spacer accumulations over time and use linear mass action kinetics to model the CRISPR reactions and the resulting ecological dynamics due to immunity and autoimmunity. Such an approach offers the computational advantage to model growing populations while simultaneously accounting for the underlying regulatory dynamics of CRISPR and its kinetics. While this model cannot capture strain-specific behavior, we can nonetheless make qualitative and even quantitative predictions for the average spacer accumulation kinetics resulting from the adaptive nature of CRISPR dynamics. The key variables in this detailed model are described in Table 2 and discussed below.

**Table 2:** Notation.

Variable	Description	Value, Units
*p*	Free cell density	*cells ml* ^−1^
*q*	Infected cell density	*cells ml* ^−1^
*v*	Phage density	*phages ml* ^−1^
(*y*_*pA*_,*y*_*pI*_,*y*_*pS*_)	Average Active, Inactive and Self-targeting spacer quota per free cell.	*spacers cell* ^−1^
(*y*_*qA*_,*y*_*qI*_,*y*_*qS*_)	Average Active, Inactive and Self-targeting spacer quota per infected cell.	*spacers cell* ^−1^
*x*_*A*_	Average Active phage protospacer quota per infected cell.	*protospacers cell* ^−1^
*α*_*p*_	Free cell replication rate	1 *hr* ^−1^
*α*_*q*_	Phage adsorption rate	5×10^−9^ *ml phage* ^−1^ *hr* ^−1^
*α*_*v*_	Phage burst size	
*γ*_*v*_	Phage death rate	5 *hr* ^−1^
Φ_*p*_	Environmental carrying capacity.	10^8^ *cells ml* ^−1^
*α*_*c*_	Acquisition rate of new spacers in Infected cells.	10^−6^ *hr* ^−1^
*γ*_*c*_	Deletion rate of new spacers in Infected cells.	varied *hr* ^−1^
*γ*_*q*→*p*_	Immunity rate per Active spacer per Infected cell.	$1-10^{-6}{\left(\frac{spacers}{cell}\right)}^{-1}\;hr^{-1}$
*γ*_*q*→*ϕ*_	Autoimmunity rate per Self-targeting spacer per Infected cell.	$\text{varied }{\left(\frac{spacers}{cell}\right)}^{-1}\;hr^{-1}$
*γ*_*q*→*v*_	Lysis rate of Infected cells	1 *hr* ^−1^
*π*_*v*_	Total number of protospacers per phage genome.	1000 *protospacers phage* ^−1^
*β*×*π*_*v*_	Total number of self-targeting protospacers per prokaryotic cell. (Defined relative to the phage protospacer level.)	varied *protospacers cell* ^−1^
*δ*	Scale factor (0 ≤ *δ≤* 1)that determines CRISPR associated reactions in free cells.	
*μ*_*v*_	Phage mutation rate per protospacer	30×10^−8^ *protospacers* ^−1^

Description of the different variables used in the detailed model. Dynamic variables are denoted with Roman letters, and parameters are denoted with Greek symbols. Any parameter associated with production of an item *i* is denoted as *α*_*i*_ and that with its degradation is denoted as *γ*_*i*_. Steady state value of item *i* will be denoted by i*. Parameter values were obtained from [30,32].

We let *π*_*v*_ denote the total number of phage protospacers per phage genome. The amount of self-targeting spacers per prokaryotic genome is defined relative to the phage protospacer amount as *βπ*_*v*_. Thus *β* = 0 implies no self-targeting protospacers per prokaryotic genome, which can also be interpreted as the absence of self-targeting protospacers due to the presence of an SND. At any time, both the free and infected cell populations (denoted as *p* and *q* respectively) have an associated CRISPR spacer content, the “per-cell” quotas which are completely specified by {*y*_*pA*_, *y*_*pI*_, *y*_*pS*_} and {*y*_*qA*_, *y*_*qI*_, *y*_*qS*_} respectively (Table 2). Here *y*_⋅*A*_ denotes the active spacer quota per cell (i.e., phage reactive), *y*_⋅*I*_ denotes the inactive spacer quota per cell (i.e., phage inactive, due to mutations in the corresponding PAMs in phages) and *y*_⋅*S*_ denotes the self-targeting spacer quota per cell. The average phage protospacer quota per infected cell available for its new spacer acquisitions is denoted by *x*_*A*_.

The per capita quotas of the various types of CRISPR spacer content are used to model the rates of acquisition and interference reactions in each subpopulation. Let *γ*_*q*→*p*_ be the rate of immunity conferred per active spacer; then at any given time the immunity rate per infected cell is assumed to be *γ*_*q*→*p*_ *y*_*qA*_. Similarly, if *γ*_*q*→*ϕ*_ denotes the rate of autoimmunity conferred per self-targeting spacer, the autoimmunity rate per infected cell is then *γ*_*q*→*ϕ*_ *y*_*qS*_. To obtain the corresponding term for the free cell population we will first need to model infection-mediated CRISPR activation.

As the operonic structure of CRISPR/*Cas* genes lends itself to regulation based on free/infected cell states ([28,29,37–40]), we simply scale the rates of all the CRISPR reactions (acquisition, deletion and interference) by *δ* (0 ≤ *δ* ≤ 1), in the free cell population relative to that of the infected population. So *δ* = 0 implies that all CRISPR reactions in free cells are switched off whereas *δ* = 1 implies that there is no differential CRISPR expression between the free and infected cell populations. Note that, only infected cells can acquire novel phage protospacers, while both infected and free cell populations can acquire self-targeting protospacers. The latter events occur when *δ* > 0. Under these modeling assumptions, the corresponding autoimmunity rate per self-targeting spacer is given by *δγ*_*q*→*ϕ*_; this is scaled by the per capita free cell quota of self-targeting spacers to calculate the autoimmunity rate per free cell, *δγ*_*q*→*ϕ*_ *y*_*pS*_.

#### Population dynamics

We now describe how the above reactions are coupled with prokaryote-phage coevolution. Free cells (*p*) replicate at a rate *α*_*p*_ under the constraint imposed by the carrying capacity Φ_*p*_. Free cells are also produced from infected cells (*q*) due to immune evasions of phage lysis at a rate of *γ*_*q*→*p*_ *y*_*qA*_ (as described above). Thus the total amount of infected cells that undergo immunity is given by *γ*_*q*→*p*_ *y*_*qA*_ *q*. Phages (*v*) infect free cells with an adsorption rate constant *α*_*q*_ to produce *q*. In addition, free cells undergo autoimmunity at a rate of *δγ*_*q*→*ϕ*_ *y*_*p*_ *S*, which is determined by the amount of self-targeting spacers (*y*_*pS*_) in free cells and the degree of CRISPR activity in free versus infected cells (*δ*). Phages with a burst size of *α*_*v*_ are produced from lysis of infected cells at rate *γ*_*q*→*v*_ and removed at a rate of *γ*_*v*_. *q* can undergo autoimmunity at a rate of *γ*_*q*→*ϕ*_ *y*_*qS*_, or switch to free cells with rate *γ*_*q*→*p*_ *y*_*qA*_. The differential equations are then given as:

$$\begin{array}{l} \dot{p}=\alpha_{p}p\left(1-\frac{p+q}{\Phi_{p}}\right)+\underset{\text{immunity}}{\underset{︸}{\gamma_{q\to p}y_{qA}q}}-\alpha_{q}pv-\underset{\text{autoimmunity}}{\underset{︸}{\delta \gamma_{q\to \phi }y_{pS}p}}\\ \dot{q}=\underset{\text{infections}}{\underset{︸}{\alpha_{q}pv}}-\gamma_{q\to p}y_{qA}q-\gamma_{q\to \phi }y_{qS}q-\underset{\text{lysis}}{\underset{︸}{\gamma_{q\to v}q}}\\ \dot{v}=\underset{\text{lysis}}{\underset{︸}{\alpha_{v}\gamma_{q\to v}q}}-\gamma_{v}v-\alpha_{q}pv \end{array}$$

For convenience in exposition below, we will let Γ_*p*_ = (*α*_*q*_ *v* + *δγ*_*q*→*ϕ*_ *y*_*pS*_) and Γ_*q*_ = (*γ*_*q*→*p*_ *y*_*qA*_ + *γ*_*q*→*ϕ*_ *y*_*qS*_ + *γ*_*q*→*v*_), which denote the overall removal rates of cells in the free and infected populations respectively.

#### Spacer and protospacer contents in free and infected cells

**Fig 3:**
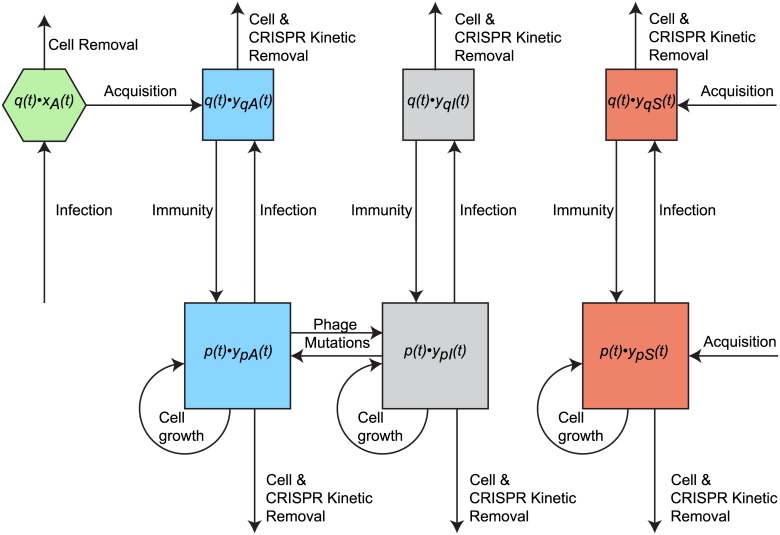
Reactions influencing total spacer and protospacer densities. The inflow and outflow of different species are indicated. The figure shows the reactions influencing the total spacer and protospacer contents at any given time in the population. We use this reaction set to derive the rates of average spacer quota change over time. Squares in the top row correspond to the total protospacer and spacer content in the infected cell population; those in the bottom correspond to those in the free cell population. Note that while we model average spacer quotas this figure illustrate all the reactions that influence *total* spacer contents.

Fig 3 presents the set of reactions influencing the total spacer and protospacer contents of different types. These give rise to the following derivatives when *q*(t) ≠ 0 and *p*(t) ≠ 0. See S1 Text for derivations.

$${\dot{x}}_{A}=\alpha_{q}\frac{pv}{q}\left[(1-\mu_{v})\pi_{v}-x_{A}\right]$$

$${\dot{y}}_{qA}=\alpha_{c}x_{A}+\;\alpha_{q}\frac{pv}{q}\left[y_{pA}-y_{qA}\right]-\gamma_{c}y_{qA}$$

$${\dot{y}}_{qI}=\alpha_{q}\frac{pv}{q}\left[y_{pI}-y_{qI}\right]-\gamma_{c}y_{qI}$$

$${\dot{y}}_{qS}=\alpha_{c}\beta \pi_{v}+\alpha_{q}\frac{pv}{q}\left[y_{pS}-y_{qS}\right]-\gamma_{c}y_{qS}$$

$${\dot{y}}_{pA}=\mu_{v}\left[y_{pI}-y_{pA}\right]+\gamma_{q\to p}\frac{y_{qA}q}{p}\left[y_{qA}-y_{pA}\right]-\delta \gamma_{c}y_{pA}$$

$${\dot{y}}_{pI}=\mu_{v}\left[y_{pA}-y_{pI}\right]+\gamma_{q\to p}\frac{y_{qA}q}{p}\left[y_{qI}-y_{pI}\right]-\delta \gamma_{c}y_{pI}$$

$${\dot{y}}_{pS}=\delta \alpha_{c}\beta \pi_{v}+\gamma_{q\to p}\frac{y_{qA}q}{p}\left[y_{qS}-y_{pS}\right]-\delta \gamma_{c}y_{pS}$$

We non-dimensionalize our equations by choosing to measure our cell density variables in units of the carrying capacity Φ_*p*_, and phage density in units of *α*_*v*_Φ_*p*_, spacer and protospacer variables in units of the number of native phage protospacers *π*_*v*_, and time in the non-dimensional units of $\tau =\alpha_{c}^{-1}t$ (CRISPR evolutionary time scales). This leads to the following set of equations, with effective parameters $A_{V}=\frac{\alpha_{v}\alpha_{q}}{\alpha_{c}}\Phi_{p}$, $G_{Q\to P}=\frac{\gamma_{q\to p}}{\alpha_{c}}\pi_{v}$, and $G_{Q\to \phi }=\frac{\gamma_{q\to \phi }}{\alpha_{c}}\pi_{v}$, while the rest of the rate parameters get scaled by $\alpha_{c}^{-1}$. Non-dimensionalization, apart from reducing the number of parameters in the model, also simplifies analysis of relative parameter sizes.

$$\begin{array}{l} \dot{P}=A_{P}\left(1-P-Q\right)+G_{Q\to P}Y_{QA}Q-A_{V}PV-\delta G_{Q\to \Phi }Y_{PS}P\\ \dot{Q}=A_{V}PV-\left(G_{Q\to P}Y_{QA}+G_{Q\to \phi }Y_{QS}+G_{Q\to V}\right)Q\\ \dot{V}=G_{Q\to V}Q-G_{V}V-\frac{A_{V}}{\alpha_{V}}PV\\ {\dot{X}}_{A}=A_{V}\frac{PV}{Q}\left[\left(1-\mu_{v}\right)-X_{A}\right]\\ {\dot{Y}}_{QA}=X_{A}+A_{V}\frac{PV}{Q}\left[Y_{PA}-Y_{QA}\right]-G_{C}Y_{QA}\\ {\dot{Y}}_{QI}=A_{V}\frac{PV}{Q}\left[Y_{PI}-Y_{QI}\right]-G_{C}Y_{QI}\\ {\dot{Y}}_{QS}=\beta +A_{V}\frac{PV}{Q}\left[Y_{PS}-Y_{QS}\right]-G_{C}Y_{QS}\\ {\dot{Y}}_{PA}=M_{V}\left[Y_{PI}-Y_{PA}\right]+G_{Q\to P}\frac{Y_{QA}Q}{P}\left[Y_{QA}-Y_{PA}\right]-\delta G_{C}Y_{PA}\\ {\dot{Y}}_{PI}=M_{V}\left[Y_{PA}-Y_{PI}\right]+G_{Q\to P}\frac{Y_{QA}Q}{P}\left[Y_{QI}-Y_{PI}\right]-\delta G_{C}Y_{PI}\\ {\dot{Y}}_{PS}\;=\delta \beta +G_{Q\to P}\frac{Y_{QA}Q}{P}\left[Y_{QS}-Y_{PS}\right]-\delta G_{C}Y_{PS} \end{array}$$ (5)

#### Simulations and bifurcation analysis

All numerical simulations were performed with Matlab 2013b. Numerical bifurcation analyses were performed with XPPAUT (AUTO) [41].

### SND absence is extremely lethal in the absence of regulation

In the absence of SND, given the large host genome size relative to that of phage (e.g. *E*.*coli* genome is roughly 100× the length of phage *λ*)and short PAM demarcating protospacers, we expect an abundant host protospacer pool. In our model, this would imply a large host to phage protospacer ratio (*β* > 1). On the other hand, if SND is present, then its efficiency determines the *β* value, with higher efficiencies implying lower *β* values and vice versa. Similarly, the parameter *δ* determines the activation level of CRISPRs in free cells relative to that of infected cells; thus *δ* = 0 represents complete repression, and *δ* = 1 signifies no difference in CRISPR activation between free and infected cell populations.

**Fig 4:**
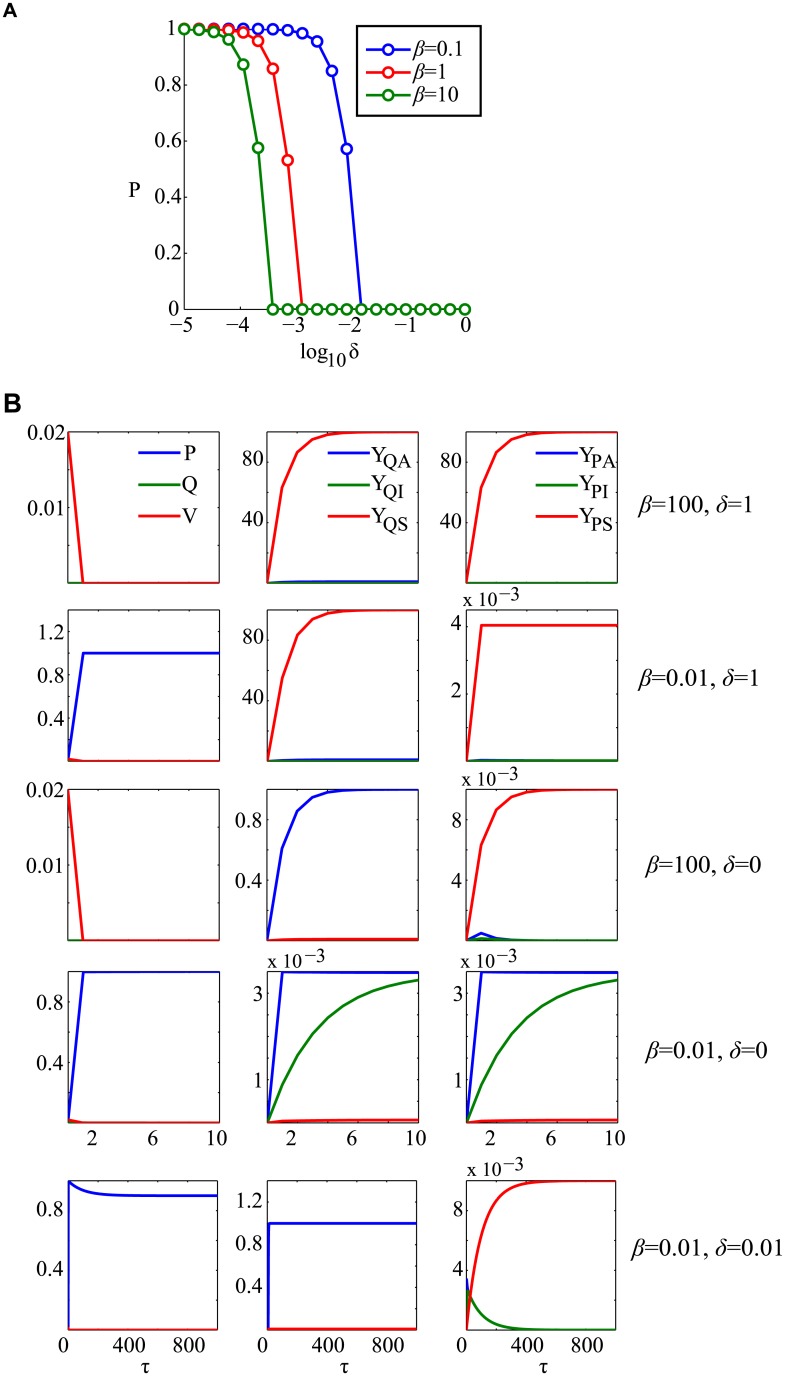
SND absence is lethal due to accumulation of self-targeting spacers. (A) A sharp threshold-like behavior is observed with steady state prokaryotic densities in the (*δ*,*β*)space. Without a sufficient amount of CRISPR suppression in free cells, determined by *δ*, cells go extinct. (B) Time course trajectories of the species and spacer variables for several parameter settings. In the absence of strong regulation of auto-immunity, high host protospacer levels are extremely toxic and cause population extinction.

To study the influence of host protospacers levels and regulation on prokaryotic densities, we vary *δ* and *β* across a large range of biologically feasible values (Fig 4). Remarkably, as we observed in the case of our simple model, the steady state prokaryotic densities show a sharp, threshold-like behavior as a function of the degree of CRISPR regulation *δ*: hosts switch from maximal densities to complete extinction as the degree of free-cell CRISPR activity, *δ*, increases (Fig 4A). Even in the case of comparable levels of host and phage protospacer (*β* = 1), greatly reduced levels of activation in free versus infected cells (*δ* < 0.01) are required to guarantee host existence. While this tight window of prokaryotic existence is relaxed slightly at lower host protospacer levels, these results indicate that tight regulatory control is necessary for a wide range of host protospacer levels. It is therefore clear that the presence or absence of an SND is a crucial determinant of CRISPR maintenance in populations.

Fig 4B shows the time course of several typical simulations for various (*β*,*δ*) combinations, to illustrate the effects of these two key parameters on intracellular spacer contents. For a wide range of parameters and initial conditions we find that the system approaches a steady state.

### A simple constraint determines CRISPR maintenance in the model

We now work to derive an analytical understanding of the critical limit on *δ* (denoted by *δ*_1_) that permits population survival. As in the simplified model, exact conditions for the threshold-like behavior of the system in the *δ* and *β* space can be obtained by considering the phage free system, in which case, the full system reduces to:

$$\begin{array}{l} \dot{P}=A_{P}P\left(1-P\right)-\delta G_{Q\to \phi }Y_{PS}P\\ {\dot{Y}}_{PA}=M_{V}\left[Y_{PI}-Y_{PA}\right]-\delta G_{C}Y_{PA}\\ {\dot{Y}}_{PI}=M_{V}\left[Y_{PA}-Y_{PI}\right]-\delta G_{C}Y_{PI}\\ {\dot{Y}}_{PS}=\delta \beta -\delta G_{C}Y_{PS} \end{array}$$

These give rise to the following fixed points: $\left\{P^{*}=1-\frac{\delta G_{Q\to \phi }Y_{PS}}{A_{P}},Y_{PA}^{*}=0,Y_{PI}^{*}=0,Y_{PS}^{*}=\frac{\beta }{G_{C}}\;\right\}$. In the absence of any feedback from infections, and in the presence of an active spacer deletion mechanism, the active and inactive spacer contents are progressively lost from the population. The influence of CRISPR induced autoimmunity on free cell density is manifest in the steady state expression for free cells. For a population to not completely lose their CRISPR activity, the condition P* > 0 must be satisfied. This leads us to the condition required for sufficient suppression of CRISPR in free cells:

$$\delta <\frac{A_{P}}{G_{Q\to \phi }Y_{PS}^{*}}=\frac{A_{P}G_{C}}{G_{Q\to \phi }\beta },$$ (6)

For values of *δ* exceeding this upper bound, the system goes extinct. The same constraint holds for a system with phage, as non-negativity of the net cellular growth rate is essential to avoid the only steady state of extinction. Note that, in the presence of a perfect SND, *β* = 1 and so the constraint on *δ* is effectively removed altogether. But in the absence of such a mechanism (*β* > 0), the internal steady state level of self-targeting spacers determines an upper limit on the free-cell CRISPR activity, *δ*.

The role of another crucial parameter is also apparent from this analysis: the *spacer deletion* rate. High spacer deletions can effectively remove self-targeting spacer accumulations, thus suppressing autoimmunity. So in addition to CRISPR regulation, the spacer deletion rate can also be increased to maintain CRISPR+ hosts in a population with larger host protospacer levels. (We will use simulations below to determine how large this rate should be relative to the spacer acquisition rate.).

### Coevolutionary dynamics under the assumption of equilibrated spacer levels over CRISPR evolutionary time scales

For a wide variety of parameters and initial conditions tested, we found that the system converged to steady states (see Fig 4B for an example). Let $\left(Y_{QA}^{*},Y_{QS}^{*},Y_{PS}^{*}\right)$ denote the resulting steady state levels of intracellular spacer contents over CRISPR evolutionary time scales. These can then determine fixed rates of immunity $\left(G_{Q\to P}Y_{QA}^{*}\right)$ and autoimmunity $\left(G_{Q\to \phi }Y_{QS}^{*},G_{P\to \phi }Y_{PS}^{*}\right)$. To do so, we use the simplified model shown in Fig 1, which replaces all immunity and autoimmunity rates (which were originally functions of the spacer variables) by fixed rate constants. In such a limit, a thorough analysis of the coevolutionary dynamics is feasible. These results indicate that as long as the constraint on *δ* is met and the steady state intracellular levels of self-targeting spacers in infected cells is non-zero, CRISPRs can exploit the abortive infection strategy alongside restriction. In the absence of SND, by contrast, the levels of self-targeting spacers will be much higher than phage reactive spacers. Under these conditions, the model predicts that CRISPRs will function principally as an abortive infection system.

We stress that we are not considering the situation that individual spacer *sequences* themselves are fixed in the population, but rather, the total number of them.

### Four characteristic regimes of CRISPR activity

Given the importance of the dimensionless parameters {*δ*,*β*,*G*_*C*_} in determining the evolutionary maintenance of CRISPR+ hosts, we now focus on understanding the influence of these parameters on the general model.

**Fig 5:**
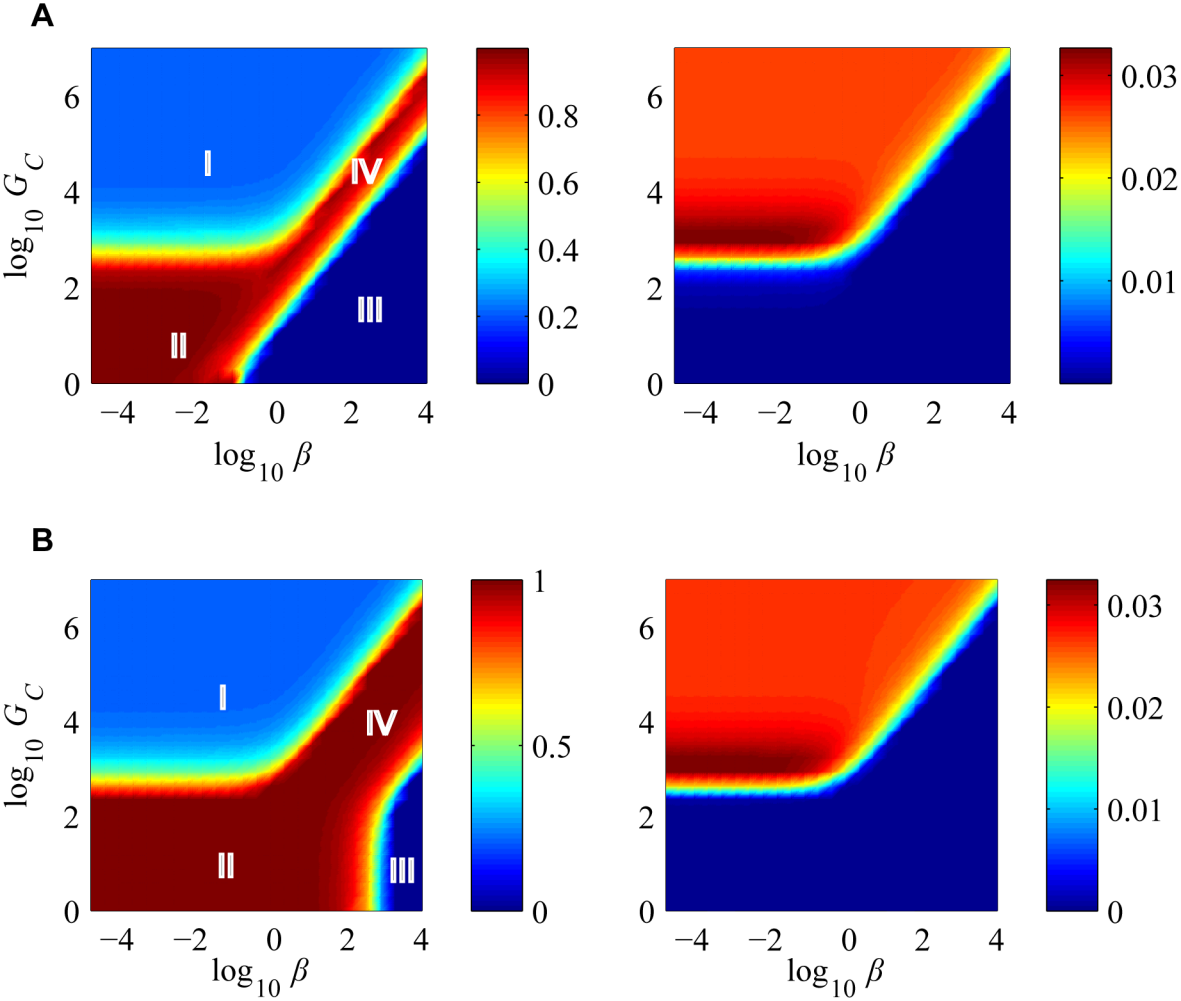
The {*δ*,*β*,*G*_*C*_} space. We plot steady state free cell densities (the first column) and the phage densities (the second column) for various values of (*β*,*G*_*C*_) values under two cases: (A) moderately suppressed free-cell CRISPR activity, *δ* = 0.01 and (B) strongly suppressed free-cell CRISPR activity, *δ* = 0.0001. *G*_*C*_ is the dimensionless parameter indicating the ratio of spacer deletion rate to spacer acquisition rate. *β* is a dimensionless parameter indicating the ratio of host to phage protospacer levels. (Regime I) Very high *G*_*C*_ values effectively reduce CRISPR content to very low levels (phage lysis rates are relatively overwhelming) offering no immune advantage to the hosts, resulting in free cell levels of $\frac{G_{Q\to V}}{A_{V}}$. (Regime II) Both abortive infection and immunity operate with the available intracellular steady state levels of active and self-targeting spacers. (Regime III) The constraint on *δ* is not satisfied and the hosts are extinct. (Regime IV) CRISPRs behave as full-fledged abortive infection systems exploiting only the accumulated self-targeting spacers, with phage reactive spacers eliminated due to high *G*_*C*_ values.

Free cell densities in the {*β*,*G*_*C*_} space for a given value of *δ* reveal a characteristic four-regime pattern. Fig 5 shows the free cell densities achieved (first column) and phage densities (second column) for various values of (*β*,*G*_*C*_) values under two cases of *δ*: *δ* = 0.01 and *δ* = 0.0001. Regime I occurs at low *β* and very high *G*_*C*_ values. Here both free cells and phages coexist; while the former assume significantly low levels (but never extinct), the latter achieve their highest densities. Regime II occurs at low *β* and low *G*_*C*_ values. Here hosts achieve their highest densities driving phage densities to very low values, if not extinction. In regime III, which occurs at high but still plausible *β* values, host extinction occurs. Regime IV is an extension of regime II’s behavior, but at high *G*_*C*_ and high *β* values.

Hints to explain the existence of these four qualitative regimes, and their boundaries, are provided by the corresponding intracellular steady state spacer levels and the constraint on *δ* we derived in the previous section. As we proceed to higher *β* values, the active spacer levels decrease and self-targeting spacer levels increase (see for example Fig 4B). Higher *β* values lead to larger steady state levels of self-targeting spacers, effectively increasing the autoimmunity rate of infected cells. This inhibits immune mediated feedback of active spacers to the free cell population (through inheritance) and causes a reduction in the overall active spacer levels. Self-targeting spacers, on the other hand, can be independently acquired in free cells at a rate determined by *δ*. According to this basic intuition, we can now derive rough conditions for falling in each of the four qualitative regimes.

(Regime I) At high *G*_*C*_ values (*G*_*C*_ *→ ∞*) CRISPR cassettes are empty and the immunity and autoimmunity reactions are overwhelmed by phage lysis. Under these conditions, both the steady state spacer levels and their derivatives become zero, making the factor $\frac{G_{Q\to P}Y_{QA}^{*}+G_{Q\to \phi }Y_{QS}^{*}}{G_{Q\to V}}=0$, resulting in no net growth advantage to CRISPR hosts (compare to $P_{C}^{*}$ steady state of the simple model). In this regime, the coevolutionary dynamics is phage limiting, resulting in steady state free cell levels of $\frac{G_{V}}{\alpha_{v}-1}$ in terms of the simple model. (Regime II) At lower *G*_*C*_ values, and when the existence condition on *δ* is satisfied, both immunity and autoimmunity operate, allowing prokaryotes to evade phage lysis at significant rates. In this regime, phages are driven to very low densities or extinction. (Regime III) At lower *G*_*C*_ values, progressing to higher *β* values increases steady-state levels of self-targeting spacers, thereby increasing the risk of not satisfying the constraint on *δ*. In such cases, regime III operates for all higher values of *β*, and extinction is inevitable. (Regime IV) This regime operates in the region where high levels of *β* are matched by corresponding high *G*_*C*_ values that are sufficient to reduce self-targeting spacer levels so as to satisfy the *δ* constraint. In this regime, host extinction occurs. Here no active spacer mediated immunity occurs, but CRISPRs transform to a full-fledged abortive infection system. When *δ* = 0, regime III does not occur, and regime IV extends into regime III. Thus the boundaries between regimes I and {II, IV} can be mapped by $\frac{G_{Q\to P}Y_{QA}^{*}+G_{Q\to \phi }Y_{QS}^{*}}{G_{Q\to V}}=0$, and that between {II, IV} and III can be mapped by the critical condition on *δ*.

### Elimination of abortive infection improves coexistence of phages

**Fig 6:**
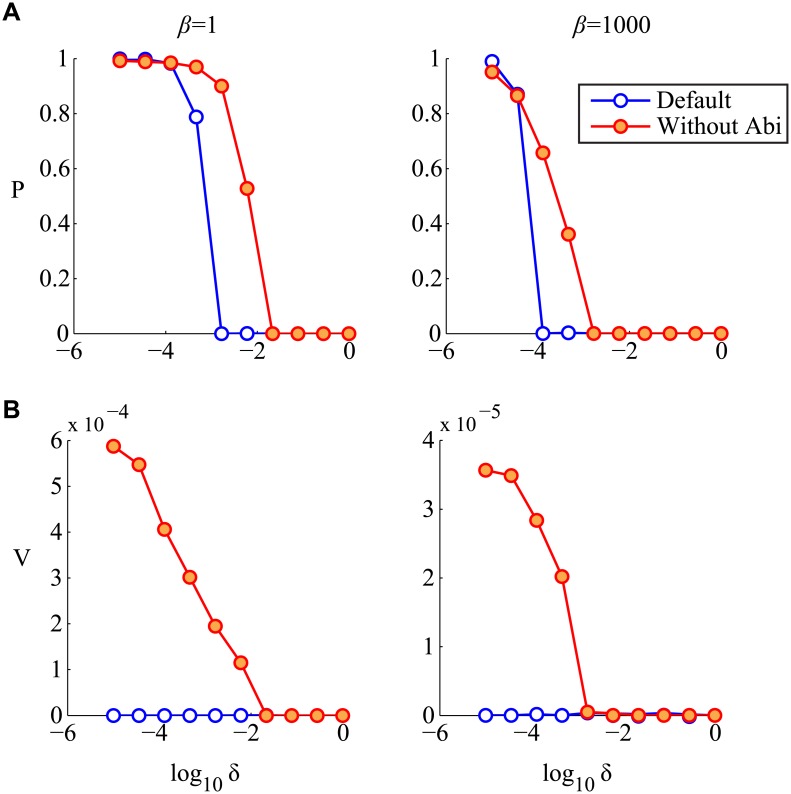
Elimination of ABI allows for improved phage densities. Steady state densities of free cells (A) and phages (B) for various values of free-cell CRISPR activity, *δ*. Both coexistence and phage densities are improved without ABI. Above a critical value of *δ*, the system goes to extinction.

To study how ABI influences the coevolutionary dynamics in the general model, we remove the autoimmunity term from the model and compare the resulting prokaryotic and phage densities across several host protospacer and CRISPR activation levels (Fig 6). We find that while removing ABI in infected cells increases the size of the coexistence regime and allows for improved phage densities. Indeed, this is the same effect predicted by our bifurcation analysis of the simplified model, where lower abortive infection rates lead to increased coexistence owing to higher phage turnover.

## Discussion

A handful of prokaryote-phage experimental systems for studying CRISPR dynamics have been established. However, the extreme diversity of CRISPRs [11] makes it difficult to draw broad conclusions from any one biological model system. Computational models, which allow exploration over a wide range of feasible parameters, provide an attractive alternative.

**Fig 7:**
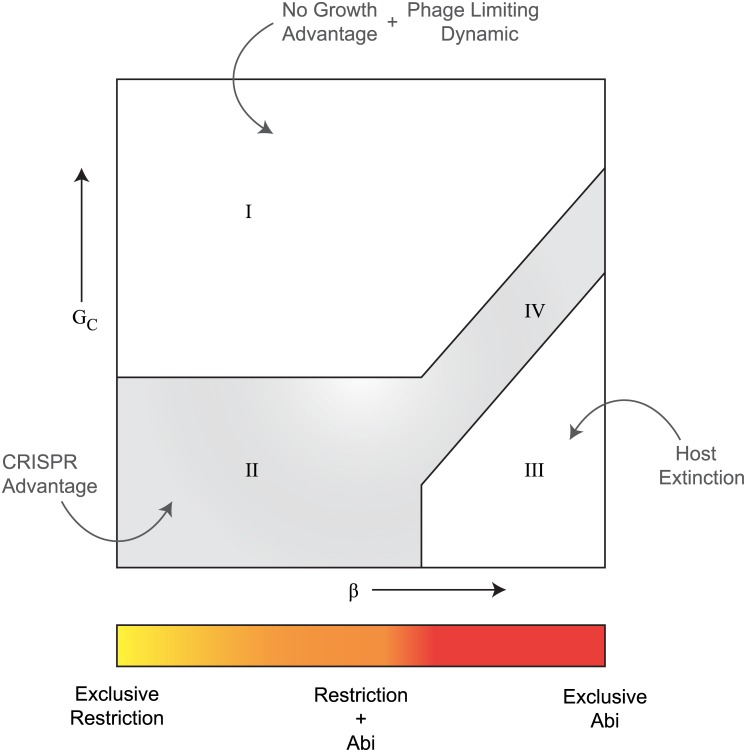
Qualitative behavior of regulated CRISPR modules. Depending on the activation level of CRISPR activity in free cells (*δ*), the host to phage protospacer ratio (*β*), and the CRISPR specific spacer deletion to acquisition rate ratio (*G*_*C*_), regulated CRISPR cassettes can fall in one of the four regimes: no advantage (regime I), advantageous to hosts by offering immune resistance and abortive infections (regime II and IV), or causing host extinction (regime III). Because per-spacer immune rates have been experimentally measured to be high, we do not study its influence specifically here. When CRISPR activity is completely repressed in free cells (*δ* = 0), regime III vanishes, and regime IV expands into its place. Notice that a low *β* value corresponds to efficient SND during acquisition process.

In this work, we analyzed the influence of infection-induced activation of CRISPRs and their autoimmunity side effect on prokaryote-phage coevolutionary dynamics. Our model integrates the classical ingredients of the prokaryotic CRISPR immune system, along with aspects of regulation and autoimmunity. Our analysis suggests that CRISPRs exploit both restriction and abortive infection. Moreover, we identified a key constraint that determines the growth advantage associated with CRISPRs as a prokaryotic immune system. As summarized in Fig 7, our model reveals a characteristic four-regime pattern determined principally by three effective parameters: the activation level of CRISPRs in uninfected population, the host to phage protospacer ratio, and spacer deletion to acquisition rate ratio in CRISPRs. In the presence of SND, the host to phage protospacer ratio is close to zero, and CRISPRs operate exclusively by exploiting restriction, while in the absence of SND, they tend to principally exploit the abortive infection route.

Several previous models have also studied CRISPR associated fitness costs, although as abstract functions. Nevertheless, these models reproduce and help to explain some of the key experimental and comparative genomics findings on CRISPRs. Levin and colleagues exploited classical density dependent ecological models to numerically analyze the invasion of costly CRISPR genotypes in the presence of innate (envelope) resistance and conjugative plasmids [20,30,42], and showed that selection due to continuous phage exposure and absence of less costly resistance mechanisms improve CRISPR maintenance in the population. Similar in spirit, Gandon and Vale make general discussions based on their analysis of general epidemiological models on the evolution of a CRISPR-*like* resistance mechanism, when the side effect associated is that of beneficial horizontal gene transfer impedance [35]. Childs et al., established a multiscale agent-based simulation model to characterize CRISPR spacer and viral diversity during coevolution, and conclude that population dynamics is more sensitive to spacer acquisition rates than interference rates [32]. Weinberger et al., derive a critical threshold on CRISPR associated cost as a function of coevolving viral diversity, innate resistance and spacer acquisition rate and conclude that high viral diversity selects against CRISPRs [34]. Iranzo et al., used numerical simulations of a general agent based simulation model that additionally accounted for CRISPR loss and horizontal transfer, to exhaustively study CRISPR maintenance as a function of various kinetic parameters in their model [33]. They also concluded that CRISPR loss is encouraged at high prokaryote/phage population sizes.

Our analyses complement these studies summarized above, and they advance our understanding of CRISPR mechanisms in general. We have delineated the precise conditions under which CRISPRs can be lost even at low viral diversities. The level of complexity in our model, intermediate to previous simulations of agent-based models and models requiring radical simplifications and that do not account for the adaptive nature of CRISPR kinetics, provides an opportunity for mathematical analysis and intuitive understanding of the results. We have presented an analytical treatment of a particular limit of our model (which empirically hold for wide parameter regimes), summarizing qualitative behavior of the CRISPR system as a function of the underlying parameters.

It is also worthwhile to re-examine previous experimental and bioinformatic studies of CRISPRs, in light of the insights gained from our modeling analyses. We found that for CRISPRs to be maintained in a population, free-cell CRISPR activity must be sufficiently suppressed. This upper bound on free-cell activity is determined by a nondimensional ratio of free cell growth rate to that of its autoimmunity potential due to the accumulated self-targeting spacers. An immediate consequence is that CRISPRs are likely to be lost from populations or cell types with reduced growth rates. This result helps to explain well-known empirical trends. For example, in general it is known that drug resistance or virulence is associated with moderate to high fitness costs; under these conditions cells often assume low growth rates [43]. According to our model, then, such strains should lack functional CRISPR elements, as has been confirmed for multi-drug resistant *Escherichia coli* [44] and for highly virulent *Francisella sp*. [45]. Furthermore, clinical isolates of *Pseudomonas aeruginosa* lack CRISPR resistance despite *crRNA* expression, and several virulent clinical isolates of pathogenic *Vibrio parahaemolyticus* [46], *Shigella* [47], pathogenic *Clostridium jejuni* [48] and *Mycoplasma gallisepticum* [49] seem to lack CRISPR resistance. While these studies have suggested a causal role played by CRISPR inactivity in the gain of virulence of clinical isolates, we propose an alternative mechanism: reduced growth rate in virulent strains induces selection for reduced CRISPR activity.

**Fig 8:**
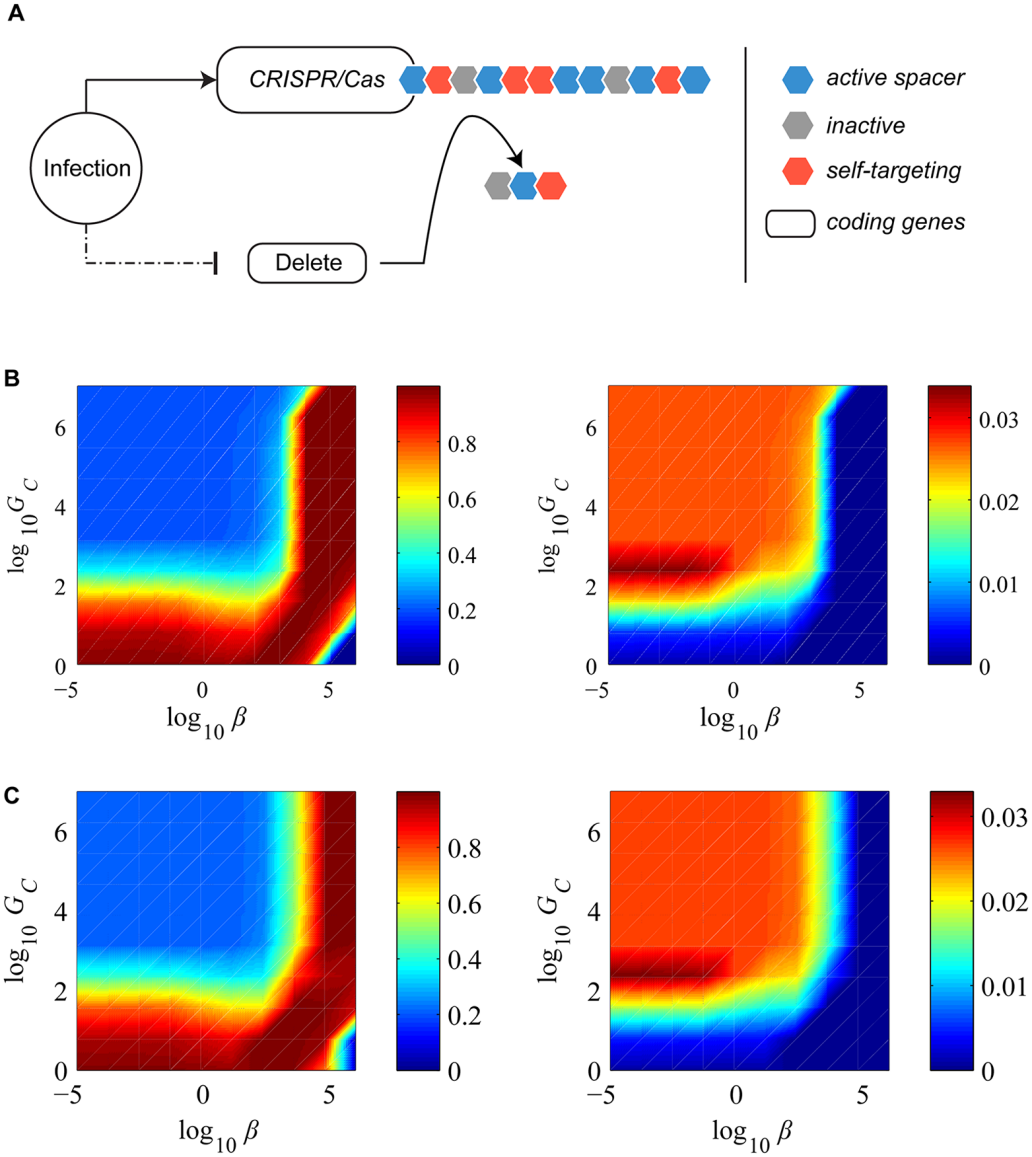
Decoupled behavior of a spacer deletion system. (A) A schematic of the decoupled model of CRISPR regulation. Arrow indicates activation, and a blunt arrow indicates repression. The dashed arrow can be active (suppression when infected) or inactive (constitutive expression). We plot the steady state free cell densities (the first column) and the corresponding phage densities (the second column) for various values of (*β*,*G*_*C*_) values at *δ* = 0.0001. Comparison with Fig 5B illustrates that decoupled spacer deletion systems as in (B) no regulation or (C) regulation in a direction opposite to that of the rest of the CRISPR system can tolerate higher host protospacer levels without requiring extremely high *G*_*C*_ values. Note that log_10_ *β* = 2 corresponds to 100× the corresponding phage protospacer levels, a realistic condition in the case of *E*.*coli* vs. phage *λ*, where the expected number of host protospacers is a hundred fold.

Under the assumptions of our model we can make approximate quantitative statements about the kinetic parameters underlying CRISPR function. In the *absence* of SND, our results suggest that CRISPRs can be maintained in a prokaryotic population only under high repression in free cells and/or high deletion rates (>10^2^ times the spacer acquisition rate in the absence of complete repression, as obtained in Fig 5). But while high repression is possible through crosstalk with specialized pathways that detect phage invasion or foreign DNA element, as is often the case with toxin/anti-toxin or abortive infection systems [28,37–40,50], how can such high deletion to acquisition ratio be achieved? One possibility is a spacer deletion mechanism [9,51–54] but we still lack sufficient biochemical characterization of this process. Our model assumed that the spacer deletion system is coupled with the rest of the CRISPR machinery, because it is likely that such a system must be expressed from the same operon as the rest of the CRISPR genes. We tested two hypothetical deletion systems that relax the requirement for high spacer deletion rates (Fig 8). The first is constitutively expressed regardless of the cell state. The second is regulated in a direction opposite to that of the rest of the CRISPR machinery—it is repressed when infected, and fully activated when uninfected. The reason these strategies work is because of the fundamental reduction they produce in the steady state expressions of the self-targeting spacers. Notice however that neither of these alterations guarantee CRISPR maintenance for arbitrarily large host protospacer levels. They still must respect the required constraint of reduced CRISPR activity in free cells.

A thorough biochemical characterization of the spacer deletion mechanism is required for advancing our understanding of CRISPRs. Stern et al. [14], in their large scale survey of CRISPR cassettes in microbial genomes, remarked that *deactivated* self-targeting spacers are found throughout the CRISPR array. This is in contrast to experimental conclusions that, in most systems, more recent acquisitions appear in the leader proximal end [51,55–57]. In fact, Stern et al. found that self-targeting spacers with no signs of deactivation were limited to the leader proximal end, indicating that their acquisition followed immediate lethality. It is therefore tempting to suggest that the spacer deletion machinery was likely impaired, resulting in continued acquisitions alongside advantageous coevolving phage targeting spacers; and the continued selection pressure to evade self-targeting activity but retain phage targeting activity persisted and selected for loss-of-function mutations in the self-targeting spacers. While this manuscript was in review, Levy et al., demonstrated that artificially induced CRISPR systems in laboratory populations of *E*.*coli* tend to exploit degradation products from the enzyme *RecBCD*, which processes double strand breaks resulting from replicating DNA and through the processing of exposed linear phage genomes after infection [58]. Because this bias reduces the effective number of self-targeting spacer acquisitions, this can be seen as a potential self- vs. non-self detection mechanism resulting in a relaxed constraint on CRISPR regulation. It is however crucial that the spacer deletion system is still in check so as to avoid the loss of effective antiviral spacers, thereby encouraging CRISPR maintenance in the population.

The rapidly growing empirical literature on CRISPR molecular and cellular biology will surely suggest further refinements to our model. Several avenues for model improvement are already apparent. First, the impact of the most commonly occurring alternative resistance mechanisms (such as envelope resistance) in laboratory populations was neglected. Second, our activation model where all CRISPR reactions are scaled uniformly in free cells is simplistic, as differences in activation levels among the acquisition and interference genes may occur. Third, assignment of equal autoimmunity rate constants for all the genomic protospacers is a rough approximation and it is known that the genetic sequences vary in their essentiality. Fourth, the current analytic cannot describe multiple CRISPR genotypes with diverse spacer configurations, in contrast to agent-based models [32,34]. Nevertheless, despite these simplifications, our analysis clarifies the effects of CRISPR autoimmunity in a general setting—a problem that is difficult to address experimentally, due to the lethality of self-targeting.

## Supporting Information

S1 Text Derivation of per cell quotas of CRISPR spacer content equations. (PDF)
