# Supplementary material for: Regulated CRISPR Modules Exploit a Dual Defense Strategy of Restriction and Abortive Infection in a Model of Prokaryote-Phage Coevolution
Source: PLoS Comput Biol. 2015 Nov 6;11(11):e1004603. doi: 10.1371/journal.pcbi.1004603 (PMC4636164; doi:10.1371/journal.pcbi.1004603)
Supplement: S1 Text — (PDF) [file pcbi.1004603.s001.pdf]

S1 Text Regulated CRISPR Modules Exploit a Dual Defense Strategy of Restriction and Abortive Infection in a Model of Prokaryote-Phage Coevolution

M. Senthil Kumar[1,2,*], Joshua B. Plotkin[3,*] and Sridhar Hannenhalli[2,*]
[1]Graduate Program in Bioinformatics, University of Maryland, College Park, MD 20742.
[2]Center for Bioinformatics and Computational Biology, University of Maryland, College Park, MD 20742.
[3]Department of Biology, University of Pennsylvania, Philadephia, PA 19104.

MSK: smuthiah@umiacs.umd.edu JBP: jplotkin@sas.upenn.edu SH: sridhar@umiacs.umd.edu

Here we derive the differential equations governing the per cell quota of CRISPR spacer contents.

Active Phage Protospacer Quota Per Infected Cell

Because we track the per capita quotas of protospacer contents per infected cell, any expression for its derivative has to account for the current spacer density in the infected cell population, the influx due to the newly infected cells, weighted by their corresponding population sizes (refer **Fig. 3**). At any time instant t, the total amount (density) of phage protospacers associated with the entire infected population is $x_A(t)q(t)$. The total amount of newly released phage protospacers is given by the product of total amount of infections and the expected amount of native phage protospacers per phage as $\alpha_q p(t)v(t)\times(1-\mu_v)\pi_v$. The total amount of protospacers leaving the infected pool is proportional to the removal rate of infected cells and is equal to $\Gamma_q(t)q(t)x_A(t)$. For any small time interval Δt then, we can write $x_A(t+\Delta t)$ as:

$$x_A(t+\Delta t)=\frac{x_A(t)q(t)+\Delta t\alpha_q(1-\mu_v)\pi_v p(t)v(t)-\Delta t\Gamma_q(t)q(t)x_A(t)}{q(t)+\Delta t\alpha_q p(t)v(t)-\Delta t\Gamma_q(t)q(t)},$$

where the denominator is the expected infected cell density at time $t+\Delta t$. Thus $x_A(t+\Delta t)$ is precisely the average protospacer content per infected cell at time $t+\Delta t$. In a straightforward fashion, when $q(t)\neq 0$, we can compute the limit $\frac{dx_A(t)}{dt}=\lim_{\Delta t\to 0}\frac{x_A(t+\Delta t)-x_A(t)}{\Delta t}$ to obtain our derivative:

$$\dot{x}_A=\frac{\alpha_q p(t)v(t)\left[(1-\mu_v)\pi_v-x(t)\right]}{q(t)}$$

We now follow a similar procedure to compute the average spacer contents in the free and infected cell populations.

CRISPR Spacer Quota Per infected Cell

We derive the derivatives for the per capita quotas of CRISPR spacer content using the same approach as above. Briefly, new additions to the active and self-targeting spacer content associated with infected cell population can occur upon infection due to acquisition reactions. In addition, they are also inherited from free cells that are infected. Inactive spacers in the infected cell population, however, can only be inherited. Furthermore, we will also account for the removal of spacers due to CRISPR kinetics through a spacer deletion parameter γ_c.

Given the current phage protospacer levels available per infected cell (x_A) for spacer acquisitions and the acquisition rate of α_c, the total amount of new active spacer acquisitions is computed as $\alpha_c x_A$. Similarly, given the current genomic protospacer density of $\beta\pi_v q$, the total amount of newly acquired self-targeting spacer content is given by $\alpha_c \beta\pi_v q$. For a given spacer type, the total inflow due to inheritance is determined by the amount of infections ($\alpha_q vp$) and the spacer density of that type in the free cell population (e.g., $\alpha_q vp \times y_{pA}$ for active spacers). Finally, all three spacer types within an infected cell are also removed at a rate proportional to the removal rate of infected cells and spacer deletion. Taken together this results in the following equations for the different spacer contents at time $t+\Delta t$:

$$y_{qA}(t+\Delta t) = \frac{q(t)y_{qA}(t) + \Delta t\alpha_c x_A(t)q(t) + \Delta t\alpha_q p(t)v(t)y_{pA}(t) - \Delta t\left(\Gamma_q + \gamma_c\right)q(t)y_{qA}(t)}{q(t) + \Delta t\alpha_q p(t)v(t) - \Delta t\Gamma_q(t)q(t)}$$

$$y_{qI}(t+\Delta t) = \frac{q(t)y_{qI}(t) + \Delta t\alpha_q p(t)v(t)y_{pI}(t) - \Delta t\left(\Gamma_q + \gamma_c\right)q(t)y_{qI}(t)}{q(t) + \Delta t\alpha_q p(t)v(t) - \Delta t\Gamma_q(t)q(t)}$$

$$y_{qS}(t+\Delta t) = \frac{q(t)y_{qS}(t) + \Delta t\alpha_c \beta\pi_v q(t) + \Delta t\alpha_q p(t)v(t)y_{pS}(t) - \Delta t\left(\Gamma_q + \gamma_c\right)q(t)y_{qS}(t)}{q(t) + \Delta t\alpha_q p(t)v(t) - \Delta t\Gamma_q(t)q(t)},$$

Computing $\lim_{\Delta t\to 0}\frac{y_{qA}(t+\Delta t) - y_{qA}(t)}{\Delta t}$, $\lim_{\Delta t\to 0}\frac{y_{qI}(t+\Delta t) - y_{qI}(t)}{\Delta t}$, and $\lim_{\Delta t\to 0}\frac{y_{qS}(t+\Delta t) - y_{qS}(t)}{\Delta t}$, for $q(t) \neq 0$, we obtain the corresponding derivatives as:

$$\dot{y}_{qA} = \frac{\alpha_c x_A(t)q(t) + \alpha_q p(t)v(t)\left[y_{pA}(t) - y_{qA}(t)\right] - \gamma_c q(t)y_{qA}(t)}{q(t)}$$

$$\dot{y}_{qI} = \frac{\alpha_q p(t)v(t)\left[y_{pI}(t) - y_{qI}(t)\right] - \gamma_c q(t)y_{qI}(t)}{q(t)}$$

$$\dot{y}_{qS} = \frac{\alpha_c \beta\pi_v q(t) + \alpha_q p(t)v(t)\left[y_{pS}(t) - y_{qS}(t)\right] - \gamma_c q(t)y_{qS}(t)}{q(t)}$$

CRISPR Spacer Quota Per Free Cell

New additions to the self-targeting spacer content in free cells is determined by the differential activation rate of acquisition in free cells ($\delta\alpha_c$), and the current available pool of genomic protospacers $\beta\pi_v p$. All three spacer types are inherited from the infected cells at a rate proportional to the amount of infected cells undergoing immunity. Further, at a rate determined by per protospacer spacer mutation rate μ_v , mutated phage protospacers can switch to being native (and vice versa); at this rate then, this effect is also reflected in the corresponding CRISPR content as the transition of inactive spacers to active states (and vice versa). For simplicity, we do not consider the difference in the rates of forward and backward mutation rates. Finally, all three spacer types within a free cell replicate at a rate proportional to the effective free cell duplication rate, and are removed at a rate proportional to the removal rate of free cells and spacer deletion (which, as mentioned before, is scaled by the CRISPR activation rate δ). Taken together this results in the following equations for average spacer contents at time $t+\Delta t$ (for clarity, we ignore mentioning time dependence explicitly):

$$y_{pA}(t+\Delta t)=\frac{\begin{array}{r} y_{pA}p+\Delta t\mu_v y_{pI}p-\Delta t\mu_v y_{pA}p+\Delta t\gamma_{q\to p}y_{qA}^2 q-\Delta t\Gamma_p p y_{pA} \\ -\Delta t\delta\gamma_c p y_{pA}+\Delta t\alpha_p p\left(1-\frac{p+q}{\Phi}\right)y_{pA}\end{array}}{p+\Delta t\gamma_{q\to p}y_{qA}q-\Delta t\Gamma_p p+\Delta t\alpha_p p\left(1-\frac{p+q}{\Phi}\right)}$$

$$y_{pI}(t+\Delta t)=\frac{\begin{array}{r} y_{pI}p+\Delta t\mu_v y_{pA}p-\Delta t\mu_v y_{pI}p+\Delta t\gamma_{q\to p}y_{qA}q y_{qI}-\Delta t\Gamma_p p y_{pI} \\ -\Delta t\delta\gamma_c p y_{pI}+\Delta t\alpha_p p\left(1-\frac{p+q}{\Phi}\right)y_{pI}\end{array}}{p+\Delta t\gamma_{q\to p}y_{qA}q-\Delta t\Gamma_p p+\Delta t\alpha_p p\left(1-\frac{p+q}{\Phi}\right)}$$

$$y_{pS}(t+\Delta t)=\frac{\begin{array}{r} y_{pS}p+\Delta t\alpha_c\beta\pi_v p+\Delta t\gamma_{q\to p}y_{qA}q y_{qS}-\Delta t\Gamma_p p y_{pS} \\ -\Delta t\delta\gamma_c p y_{pS}+\Delta t\alpha_p p\left(1-\frac{p+q}{\Phi}\right)y_{pS}\end{array}}{p+\Delta t\gamma_{q\to p}y_{qA}q-\Delta t\Gamma_p p+\Delta t\alpha_p p\left(1-\frac{p+q}{\Phi}\right)}$$

Calculating $\lim_{\Delta t\to 0}\frac{y_{pA}(t+\Delta t)-y_{pA}(t)}{\Delta t}$, $\lim_{\Delta t\to 0}\frac{y_{pI}(t+\Delta t)-y_{pI}(t)}{\Delta t}$, and $\lim_{\Delta t\to 0}\frac{y_{pS}(t+\Delta t)-y_{pS}(t)}{\Delta t}$, $p(t)\neq 0$, we obtain the corresponding derivatives as:

$$\dot{y}_{PA}=\mu_v\left[y_{pI}-y_{pA}\right]+\gamma_{q\to p}\frac{y_{qA}q}{p}\left[y_{qA}-y_{pA}\right]-\delta\gamma_c y_{pA}$$

$$\dot{y}_{PI}=\mu_v\left[y_{pA}-y_{pI}\right]+\gamma_{q\to p}\frac{y_{qA}q}{p}\left[y_{qI}-y_{pI}\right]-\delta\gamma_c y_{pI}$$

$$\dot{y}_{qS}=\delta\alpha_c\beta\pi_v+\gamma_{q\to p}\frac{y_{qA}q}{p}\left[y_{qS}-y_{pS}\right]-\delta\gamma_c y_{pS}$$
